# The Transcription Factor CaNAC81 Is Involved in the Carotenoid Accumulation in Chili Pepper Fruits

**DOI:** 10.3390/plants14142099

**Published:** 2025-07-08

**Authors:** Maria Guadalupe Villa-Rivera, Alejandra Castañeda-Marín, Octavio Martínez, Neftalí Ochoa-Alejo

**Affiliations:** 1 Departamento de Ingeniería Genética, Centro de Investigación y de Estudios Avanzados del Instituto Politécnico Nacional, Irapuato 36824, Guanajuato, Mexico; gvillarivera@gmail.com (M.G.V.-R.); ale.castaneda@cinvestav.mx (A.C.-M.); 2 Unidad de Genómica Avanzada, Centro de Investigación y de Estudios Avanzados del Instituto Politécnico Nacional, Irapuato 36824, Guanajuato, Mexico; octavio.martinez@cinvestav.mx

**Keywords:** *CaNAC81*, *Capsicum annuum*, transcriptional regulation, carotenoid biosynthesis

## Abstract

During fruit ripening in *Capsicum* species, substantial amounts of carotenoids accumulate in the pericarp. While the carotenoid biosynthesis pathway in *Capsicum* species has been extensively investigated from various angles, the transcriptional regulation of genes encoding carotenoid biosynthetic enzymes remains less understood in this non-climacteric horticultural crop compared to tomato, a climacteric fruit. In the present study, we investigated the function of the *NAM*, *ATAF1/2* or *CUC2 81* (*CaNAC81*) transcription factor gene. This gene was selected through RNA-Seq co-expression analysis based on the correlation between expressed transcription factor gene profiles and those of carotenoid structural genes. To determine its role in regulating the expression of biosynthetic-related carotenogenic genes, we performed Virus-Induced Gene Silencing (VIGS) assays in the Serrano-type *C. annuum* ‘Tampiqueño 74’. Fruits from plants infected with a pTRV2:*CaNAC81* construct (silenced fruits) exhibited altered carotenoid pigmentation accumulation, manifested as yellow-orange spots, in contrast to fruits from non-agroinfected controls (NTC) and fruits from plants infected with the empty TRV2 construct (red fruits). Quantitative real-time PCR (qPCR) assays confirmed decreased transcript levels of *CaNAC81* in fruits displaying altered pigmentation, along with reduced transcription of the *PSY* gene, which encodes the carotenoid biosynthetic enzyme phytoene synthase (PSY). High-performance liquid chromatography (HPLC) analysis revealed a distinct carotenoid pigment accumulation pattern in fruits from plants showing silencing symptoms, characterized by low concentrations of capsanthin and zeaxanthin and trace amounts of capsorubin, compared to control plants (NTC). These findings suggest the involvement of *CaNAC81* in the regulatory network of the carotenoid biosynthetic pathway in chili pepper fruits.

## 1. Introduction

Chili pepper (*Capsicum* genus, belonging to the Solanaceae family) is a significant horticultural crop cultivated worldwide. The *Capsicum* genus encompasses 38 wild and domesticated species, among which, *C. annuum*, *C. baccatum*, *C. chinense*, *C. frutescens* and *C. pubescens*, are cultivated [1]. Chili pepper fruits are consumed fresh or dried and serve various purposes such as a spice, vegetable or in traditional medicine [2]. Furthermore, important pharmacological applications have been reported for chili pepper fruits; in vitro studies have demonstrated their antioxidant, antibacterial, antiviral, antiproliferative, anti-adipogenic, antimutagenic, and enzyme inhibitory activities. Moreover, in vivo studies have shown that *Capsicum* spp. fruits possess anti-inflammatory, hepatoprotective, antidiabetic, renoprotective, hypocholesterolemic, antitumor, anti-obesity, analgesic and gastric acid secretory activities [3]. These nutraceutical properties of chili pepper are associated with its rich composition of bioactive compounds, including carotenoids (provitamin A), vitamin C and E, capsaicinoids, flavonoids, minerals, and phenolic compounds such as quercetin and luteolin [4,5].

**Figure 1:**
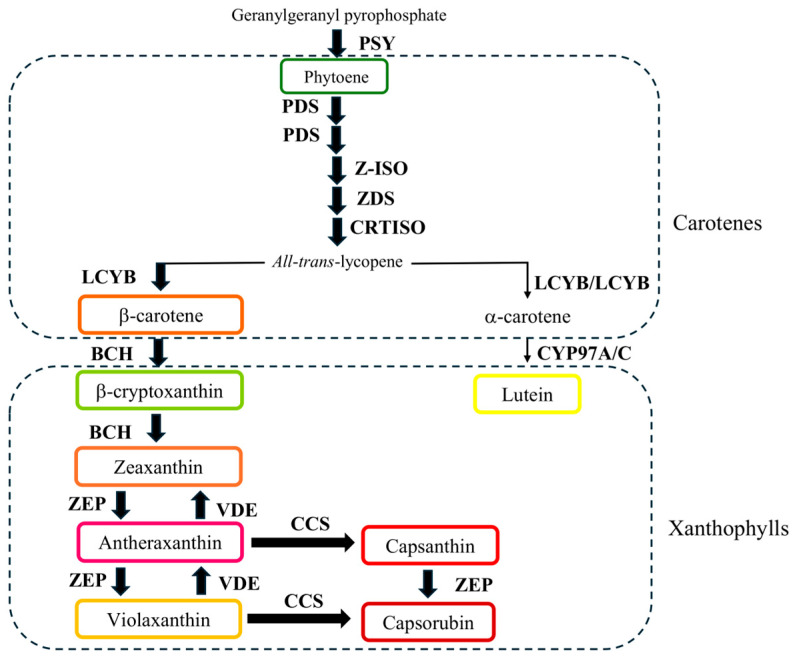
Carotenoid biosynthetic pathway in chili pepper fruits. Phytoene synthase (PSY), phytoene desaturase (PDS), ζ-carotene isomerase (Z-ISO), ζ-carotene desaturase (ZDS), carotene isomerase (CRTISO), lycopene-β-cyclase (LCYB), lycopene-ε-cyclase (LCYE), β-carotene hydroxylase (BCH), carotene hydroxylase cytochrome 450 type (CYP97 A/C), zeaxanthin epoxidase (ZEP), violaxanthin de-epoxidase (VDE), capsanthin/capsorubin synthase. Modified from [8].

Carotenoids are isoprenoid compounds with backbones composed of 40 carbon atoms. Based on their chemical composition, they are classified into carotenes (hydrocarbon structure) and xanthophylls (which contain oxygen atoms in their hydrocarbon backbones) [6,7]. Notably, the diverse array of carotenoids present in chili pepper fruits gives rise to their characteristic yellow, orange, and red colorations [8,9]. Specifically, the intensity of red color in chili pepper fruits is associated with the accumulation of capsanthin and capsanthin esters within the chromoplasts of the pericarp tissue during the ripening process [10]. In *Capsicum* fruits, carotenoids are biosynthesized via the methylerythritol phosphate (MEP) pathway, utilizing pyruvate and glyceraldehyde 3-phosphate to produce isopentenyl pyrophosphate (IPP) and dimethylallyl pyrophosphate (DMAPP). The condensation of three IPP molecules with one DMAPP molecule yields geranylgeranyl pyrophosphate (GGPP), the direct precursor of the carotenoid biosynthesis pathway (Figure 1) [11].

The initial step of the carotenoid biosynthetic pathway involves the condensation of two molecules of GGPP, catalyzed by the phytoene synthase enzyme (PSY), to produce phytoene. Subsequently, a series of desaturation and isomerization reactions, carried out by phytoene desaturase (PDS), ζ-carotene isomerase (Z-ISO), ζ-carotene desaturase and carotene isomerase (CRTISO), leads to the formation of all-*trans*-lycopene. The following steps involve the cyclization of lycopene to synthesize diverse carotenoids; these reactions are catalyzed by lycopene β-cyclase (LCYB) and lycopene ε-cyclase (LCYE). Subsequent hydroxylation of α and β-carotene, mediated by carotene hydroxylases (BCH and CYP97A/C), generates lutein and zeaxanthin. Next, zeaxanthin undergoes an epoxidation reaction, catalyzed by zeaxanthin epoxidase (ZEP), to produce antheraxanthin and violaxanthin. The reverse reaction, the conversion of violaxanthin to antheraxanthin, is catalyzed by violaxanthin de-epoxidase (VDE). Finally, capsanthin and capsorubin are produced in chili pepper fruits from antheraxanthin or violaxanthin through a reaction catalyzed by capsanthin/capsorrubin synthase (CCS) (Figure 1) [11,12]. Carotenoid accumulation in chili pepper fruits occurs during the ripening process and is stimulated by light absorbed by chloroplasts, which leads to the biogenesis of chromoplasts, the organelles where carotenoid biosynthesis and its accumulation take place [13,14]. Nitrogen fertilization is another abiotic factor influencing carotenoid biosynthesis; reduced nitrogen availability has been reported to increase carotenoid accumulation in chili pepper fruits [15]. At the molecular level, the carotenoid biosynthetic pathway can be regulated through metabolite feedback, as well as transcriptional and epigenetic mechanisms [16]. While phytohormones and transcription factors (TFs) involved in the transcriptional regulation of this pathway have been extensively studied in tomato fruit, fewer studies have focused on the transcriptional and epigenetic regulation of carotenoid biosynthesis in chili pepper fruits. Recent sequencing technologies have facilitated the identification of putative TF candidates that may regulate this pathway in *Capsicum* spp. Analyses of chili pepper genomic and transcriptomic data have identified potential TF candidates possibly involved in the regulation of carotenoid biosynthesis in chili pepper fruits; for example, genes encoding ethylene responsive transcription factors (ERF) subfamily members such as *CaERF66*, *CaERF82*, *CaERF97*, *CaERF101*, and *CaERF107*, showed expression patterns similar to the accumulation profiles of β-carotene, zeaxanthin, and capsorubin levels in the pericarp of chili pepper fruits [17]. Similarly, *CaDIV1* and *CaMYBR-5* genes exhibited consistent co-expression patterns with that of the *CCS* gene in *Capsicum* spp. [18]. Moreover, RNA-Seq data identified differentially expressed genes encoding TFs such as CONSTANS-LIKE 9, GATA transcription factor 26, F-box protein SKIP23, FYVE/PHD-type, RING/FYVE/PHD-type, U-box domain-containing protein 52, and zinc finger family as possible regulators of *CCS* gene expression [19]. Furthermore, expression analysis of five TF genes belonging to the *bHLH* family (*CabHLH009*, *CabHLH032*, *CabHLH048*, *CabHLH095* and *CabHLH100*) revealed a high correlation with the accumulation patterns of lutein, zeathantin, and capsorubin in chili pepper fruits [20]. Likewise, 54 putative candidates associated with the regulation of the carotenoid biosynthesis pathway were suggested by RNA-Seq co-expression analyses and also supported by the presence of putative TF binding sequences in the promoters of genes encoding carotenogenic enzymes [21]. Additionally, the construction of gene functional networks allowed the selection of 16 TFs (belonging to the MADS, MYB, bZYP, bHLH, NAC, and ERF families) as candidates to regulate the carotenoid biosynthesis pathway (six in fruit and ten in flower) in *Capsicum* spp., narrowing down the initial list of the proposed candidates [22]. On the other hand, experimental evidence has confirmed the role of two TFs in the regulation of this pathway in *C. annuum* L. Knockdown of *CaMYB306* resulted in a significant decrease in the expression levels of genes related to the carotenogenic pathway, leading to a reduction in the accumulation of these pigments in chili pepper fruits [23]. Finally, silencing the *CaBBX20* gene in chili pepper reduced carotenoid accumulation and decreased the expression levels of *CaCCS*, suggesting its regulatory role in carotenoid biosynthesis [24].

NAM, ATAF1/2, and CUC2 (NAC) constitute a family of transcription factor (TF) genes expressed across various developmental stages and tissues of plants [25]. Members of the NAC family have been implicated in diverse functions in chili pepper, including defense responses [26], tolerance to biotic and abiotic stress [27,28,29,30,31,32,33], ripening [34], and the regulation and accumulation of carotenoids [15,35]. In *C. annuum* L., 104 genes encoding NAC TFs have been identified and categorized into three phylogenetic clusters, with one cluster containing *CaNAC* genes specific to the Solanaceae family [36]. In the present study, we evaluated the role of *CaNAC81* in carotenoid accumulation in chili pepper fruits. Our hypothesis is that this TF participates in the transcriptional regulation of genes encoding enzymes involved in carotenoid biosynthesis.

## 2. Results

### 2.1. Selection of the CaNAC81 TF Gene

Previously in [21], using RNA-Seq data obtained from *C. annuum* fruits (12 different accessions (four wild, six domesticated, and two reciprocal crosses between a wild and a domesticated accession)) during fruit developmental and ripening stages (flower (0), 10, 20, 30, 40, 50, and 60 days after anthesis (DAA), coexpression assays conducted in the g2g.TF candidate function (available in the R version 4.3.2 package *Salsa* 1.0) suggested the NAC domain-containing protein 72-like (NAC72) as a potential TF candidate for regulating the expression of the *phytoene synthase 2* (*PSY2*) gene. Furthermore, in [22], gene functional co-expression networks, constructed with the “Gene2TF” and “Gene2Gene” algorithms (of the R version 4.3.2 package *Salsa* 1.0), also highlighted NAC72 as a potential TF candidate for regulating carotenoid-biosynthesis-related genes (*PSY*, *CRTISO2*, *BCH*, and *CCS*) because they possess putative *cis*-regulatory elements in their promoters.

Amino acid sequence comparison of NAC72 (NCBI identifier XP_016569664.2) using the Pepper Genome Platform (PGP) http://peppergenome.snu.ac.kr/blast.php (accessed on 7 November 2024), revealed that this sequence corresponded to *Capana09g000936* of the *C. annuum* var. Zunla-1 annotation and *Capang00g000431* of *C. annuum* var. *glabriusculum* annotation. Using these annotation identifiers, it was possible to determine that the TF initially identified as NAC72 actually corresponded to CaNAC81, according to the genome-wide analyses of the NAC TF family of chili pepper [36]; therefore, this identification was adopted hereafter. Additionally, the comparison of the CaNAC81 amino acid sequence in the Pepper genome sequence and database of variants (Pepper GD) (http://ted.bti.cornell.edu/cgi-bin/pepper/index, consulted on 10 June 2025) [37] allowed the association of CaNAC81 with additional gene identifiers: *Caz09g09180.1* from *Capsicum annuum* var. *annuum* in the Annuum clade, and *Cbp03g24500.1* belonging to *C. baccatum* var. *pendulum* and *C. pubescens* in the Pubescens clade.

**Figure 2:**
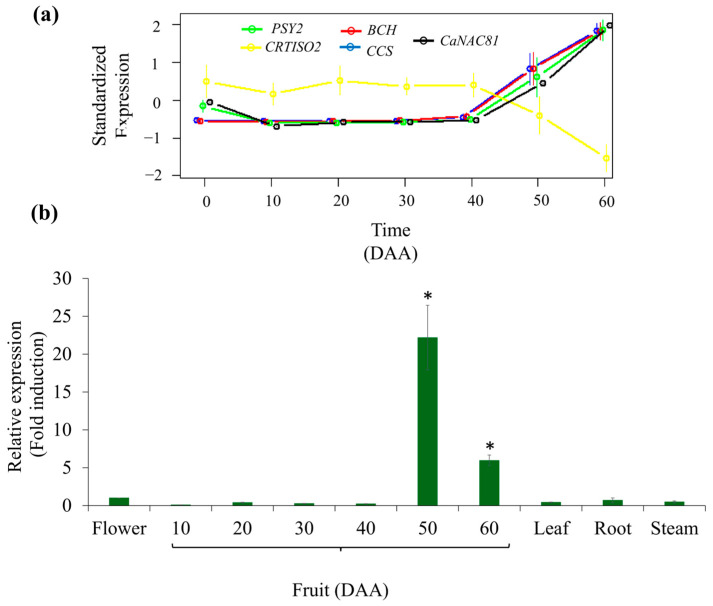
Expression pattern of *CaNAC81*. (**a**) Standardized Expression Profile (SEP) from RNA-Seq data of *CaNAC81* and proposed target genes phytoene synthase 2 (*PSY2*), carotene isomerase (*CRTISO2*), β-carotene hydroxylase (*BCH*), and capsanthin/capsorubin synthase (*CCS*) during the growth and ripening of *C. annuum* Serrano ‘Tampiqueño 74’ fruits. (**b**) Analyses of *CaNAC81* expression in flower, fruit, leaf, root and stem tissues of *C. annuum* ‘Tampiqueño 74’ conducted with qPCR. Each bar shows the average of three biological replicates ± standard deviation using ΔΔ*C*_T_ method for data transformation. “*” indicates significant changes (*p* < 0.05).

The expression profiles of *CaNAC81* (candidate gene) and the carotenoid-biosynthesis-related genes *PSY*, carotene isomerase *CRTISO2*, *BCH* and *CCS* (target genes; TGs) are summarized in Figure 2a; the expression values are presented as the Standardized Expression Pattern (SEP), as was previously reported in [38]. Figure 2a shows that *CaNAC81* and the target genes *PSY2*, *BCH*, and *CCS* exhibited similar expression profiles, with no or low transcript accumulation from 0 to 40 days after anthesis (DAA). During 50 and 60 DAA, an increase in expression levels was observed, coinciding with color changes in chili pepper fruits due to carotenoid synthesis and accumulation. In contrast, the expression of *CRTISO2* showed higher transcript levels compared to *CaNAC81* and the other TGs during the first 40 DAA followed by a decrease in the subsequent ripening stages (Figure 2a).

Furthermore, the expression levels of *CaNAC81* in chili pepper ‘Tampiqueño 74’ were corroborated by quantitative PCR (qPCR) (Figure 2b) conducted in flower, fruits at 10, 20, 30, 40, 50, and 60 DAA, leaf, root, and stem tissues. The pattern of transcripts accumulation during growth and ripening stages resembled the Standardized Expression Pattern (SEP) profile obtained from RNA-Seq assays in flower and fruits at 0–50 DAA. However, at 60 DAA, a decrease in expression levels was detected, which differed from the SEP pattern. Additionally, lower levels of transcript accumulation were observed in stem, leaf, and root tissues. Analysis of variance (ANOVA) showed significant differences (*p* < 0.01), and the post hoc Tukey test revealed that the expression levels were significantly higher (*p* < 0.05) in fruits at 50 and 60 DAA.

**Figure 3:**
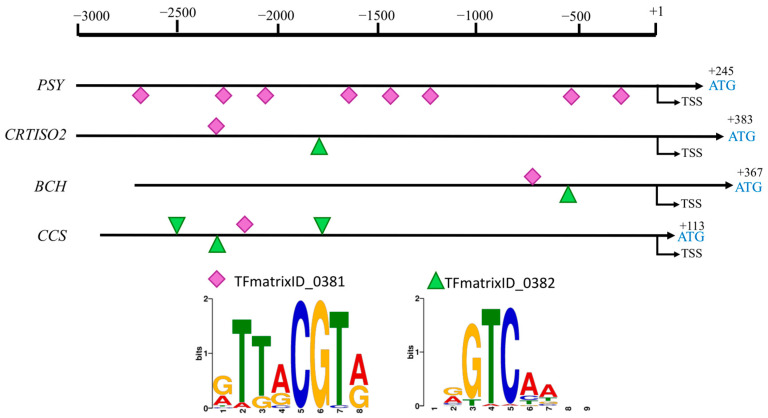
Location of putative binding sites for CaNAC81 TF in *PSY2*, *CRTISO2*, *BCH*, and *CC* promoters and their putative union sequences.

Furthermore, the promoter regions of the target genes (TGs) were analyzed following the methodology reported in [21] and using the *Arabidopsis thaliana* ortholog of *CaNAC81*, *AT4G27410*, along with two binding consensus sequences reported in The Plant Promoter Analysis Navigator (PlantPAN) (Figure 3). The *PSY2* promoter contained the highest number of consensus recognition sequences for CaNAC81 (eight putative binding sites), while the *CCS* promoter had four binding sites, and *CRTISO2* and *BCH* each had two sites (Figure 3, Supplementary Table S1). Based on this analysis, *CaNAC81* was selected for further gene function studies in this work.

### 2.2. Characterization of the CaNAC81 TF Gene

**Figure 4:**
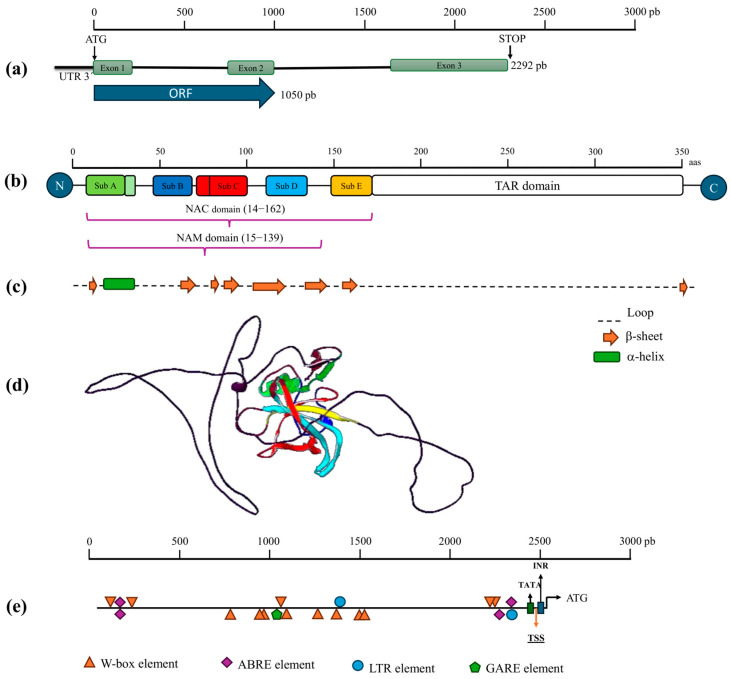
(**a**) Structure of *CaNAC81* gene, Open Reading Frame (ORF). (**b**) Domains and subdomains (Sub) of CaNAC81, and Transcriptional Activator Region (TAR). (**c**) CaNAC81 secondary structure prediction. (**d**) Protein homology modeling of CaNAC81 showing five subdomains of NAC domain and TAR domain. (**e**) Localization of putative binding sites in the *WRKY81* transcription factor gene promoter for the defense response (W-box), abscisic acid responsive element (ABRE), low-temperature-responsive element (LTR), and gibberellin-responsive element (GARE).

The genomic sequence of *CaNAC81* is located within the locus *VYZY01017293* (region 3540-531) of the *C. annuum* cultivar ECW scaffold192042. The *CaNAC81* gene spans 2292 base pairs (pb) in length, and AUGUSTUS gene prediction analysis confirmed that it comprises three exons separated by two introns, with a coding sequence of 1050 pb (Figure 4a) (Supplementary Figure S1). The deduced amino acid (aa) sequence of the *CaNAC81* gene is 350 in length. Comparison of the protein sequence against the InterPro 103.0 database for domain prediction confirmed that CaNAC81 contains the NAM domain (aa 15–139) as part of the integrated NAC domain (aa 14–162) and a Transcriptional Activation Region (TAR) at the C-terminus. Motif analysis performed on the NAC domain revealed that CaNAC81 possesses seven characteristic motifs grouped into five subdomains (Sub A, B, C, D, and E) (Figure 4b).

Through a comparison of the CaNAC81 amino acid sequence in the Plant Transcription Factors Data Base (PlantTFDB), it was possible to identify the orthologous of *CaNAC81* in *Solanum lycopersicum* (*Solyc12g013620*), corresponding to *SlNAC090*, and in *A. thaliana* (*AT4G27410*, as mentioned previously).

Using the *CaNAC81* protein sequence, the secondary structure was predicted with the Jpred 4 Server (Figure 4c). The results indicated that the NAC domain of CaNAC81 is composed of one α-helix and seven β-sheets. Only one β-sheet structure, seemingly formed with irregular loop structures, was predicted in the TAR domain. Additionally, homology modeling of the CaNAC81 tridimensional (3D) structure was performed using the SWISS-MODEL Server (Figure 4d). A total of 47 templates were identified, but the NAC domain-containing protein JA2 AlphaFold DB model from *S. lycopersicum* (PDB code: Q9SQL0-1A), with a sequence identity of 89.6% and a coverage of 0.99, was selected as the template for predicting the CaNAC81 3D structure. The quality of the predicted model was assessed by the Global Model Quality Estimate (GMQE), QMEANDisco global score, and MolProbability Score, yielding values of 0.62, 0.43 ± 0.05, and 1.79, respectively. Energy minimization of the TF model resulted in a value of −13,196.090 kJ/mol. Finally, a Ramachandran plot showed that 78.74% of residues were located in the allowed region, with 11.21% as outliers.

Analysis of the *CaNAC81* promoter revealed a 2500 bp intergenic region in the genomic sequence of the locus *VYZY01017293* from *C. annuum* cultivar ECW scaffold192042. To define the promoter region, consensus sequences for the TATA box (TATATAAATCCC), Initiator element (INR), and the putative Transcription Start Site (TSS) at position 2448 were predicted using the YAPP Eukaryotic Core Promoter Predictor tool, yielding the highest combined score of 1.94. Further analysis of the *CaNAC81* promoter was performed using the New PLACE database, identifying four putative binding sites for a *cis*-acting element involved in abscisic acid responsiveness (ABRE), one motif for a gibberellin-responsive element (GARE), two putative binding sites for a low-temperature-responsive element (LTR), and thirteen motifs for the defense response (W-box) (Figure 4d). The location of each putative binding site and the corresponding consensus sequences are summarized in Supplementary Table S2.

Moreover, phylogenetic analyses using the amino acid sequences of CANAC81 and other NAC TFs previously reported as regulators of carotenoid accumulation in chili pepper and tomato were performed. Multiple sequence alignment (MSA) of protein sequences of SNAC 4, SNAC 9 and NOR-like 1 from *S. lycopersicum* [39,40], and its corresponding orthologous in chili pepper, as well as the NAC TF identified as CA12g04950, reported as a regulator of accumulation of pigments in chili pepper in response to nitrogen reduction [15], was also conducted. Additionally, the sequence of CaNAC72 reported in [36] was included. The MSA revealed conserved motifs corresponding to the NAC domain at the N-terminus, and a high variation at the TAR domain was observed (Supplementary Figure S2a).

The phylogenetic analyses of NAC TFs showed similar topologies using the Neighbor-Joining or Maximum Likelihood methods (Supplementary Figure S2b). The topology showed that genes of tomato CNAC 4, 9 and NOR-like 1 were clustered with its chili pepper ortholog, while CaNAC81 and CaNAC72 were clustered together. The sequence corresponding to CA12g04950 was located as basal of the rest of the NAC TFs. These results confirmed that CaNAC81 represents a novel candidate to be evaluated as a regulator of carotenoid accumulation in chili pepper.

### 2.3. Virus-Induced Silencing of CaNAC81 in Chili Pepper

**Figure 5:**
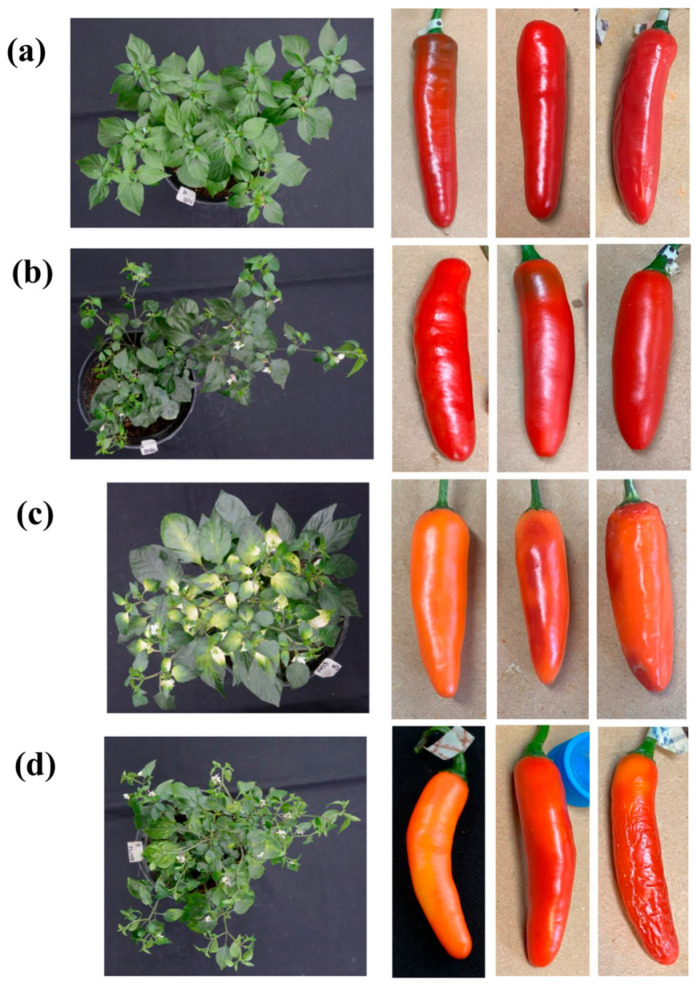
Virus-induced silencing assays of *CaNAC81* gene in chili pepper plants. Plants infiltrated and fruits collected from plants infiltrated with (**a**) sterile distilled water as negative control (NTC), (**b**) empty vector TRV2, (**c**) construct TRV2:*PDS* as positive control of agroinfiltration, and (**d**) construct TRV2:*CaNAC81*.

To investigate the function of the *CaNAC81* TF gene as a possible regulator of carotenoid-biosynthesis-related structural genes, Virus-Induced Gene Silencing (VIGS) assays were performed. Figure 5 displays representative images of chili pepper plants infiltrated with sterile distilled water (negative control (NTC)), plants agroinfiltrated with the empty TRV2, plants agroinfiltrated with the TRV2:*PDS* construct (positive control of agroinfiltration), and plants agroinfiltrated with TRV2:*CaNAC81* constructs, respectively. Additionally, representative fruits collected from these plants are shown in Figure 5.

**Figure 6:**
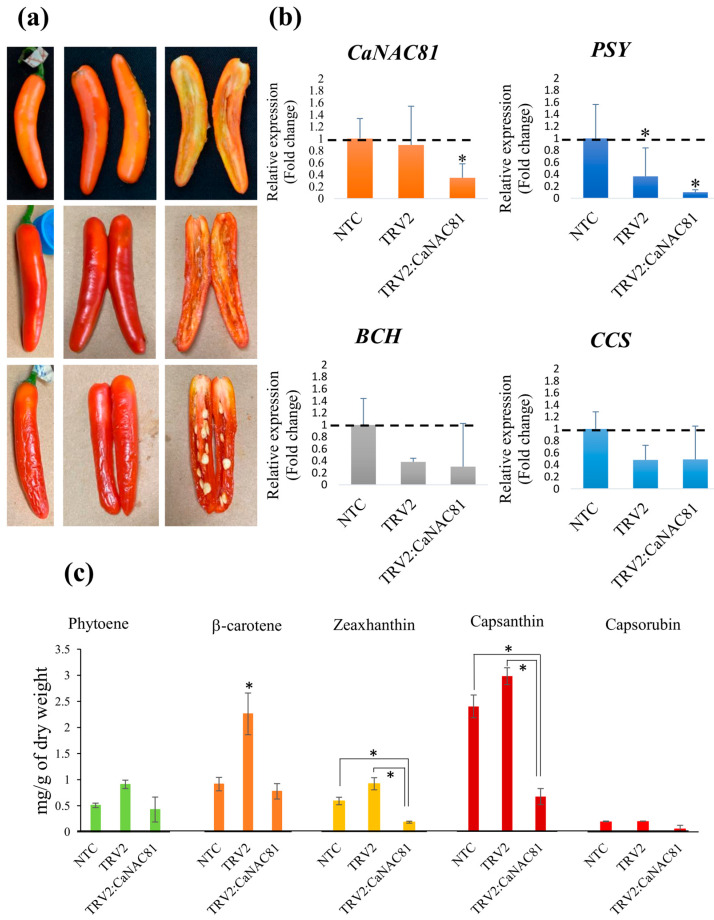
Analyses of fruits from TRV2:*CaNAC81* agroinfiltrated plants. (**a**) External and inner fruit parts of three biological replicates collected from plants agroinfiltrated with the TRV2:*CaNAC81* construct showing delimited yellow-orange patches. (**b**) Quantitation by qPCR of *CaNAC81, PSY, BCH* and *CCS* transcript accumulation in fruits collected from plants treated with the TRV2:*CaNAC81* construct. Graphics were generated by data transformation using the ΔΔC_T_ method. Each bar shows the mean of three biological replicates ± SD. Asterisks indicate statistically significant changes. (**c**) Quantitation of carotenoids in fruits from control plants (NTC) and agroinfiltrated plants (TRV2 and TRV2:*CaNAC81*). Concentration is expressed as mg/g of dry weight. Each bar shows the mean of concentration of three biological replicates ± SD. “*” indicates significant changes (*p* < 0.05).

Five plants from each treatment group were grown and maintained until fruit harvest. As anticipated, no phenotypic changes were observed in plants and fruits from the negative control (NTC) plants (Figure 5a). However, characteristic symptoms of viral infection were evident in the leaves of plants agroinfiltrated with the empty TRV2 vector and TRV2:*CaNAC81* (Figure 5b,d). Additionally, the positive control plants agroinfiltrated with the *PDS* construct exhibited phenotypic changes (white patches) in their leaves, indicating the successful silencing of the *PDS* gene (Figure 5c). At least three fruits (biological replicates) were collected from each treatment group. Alterations in color accumulation were detected in fruits from plants agroinfiltrated with TRV2:*PDS* and TRV2:*CaNAC81*. Fruits collected from the TRV2:*PDS* positive control plants showed significant changes in color accumulation compared to the NTC and TRV2 controls, displaying a pale orange color with small red patches (Figure 5c). Interestingly, fruits from plants agroinfiltrated with TRV2:*CaNAC81* exhibited delimited yellow-orange patches (Figure 5d and Figure 6a), suggesting a differential accumulation of carotenoids in comparison with the red fruits of the NTC group at 50–60 DAA. The pericarp tissue was dissected from these fruits, immediately frozen in liquid nitrogen, and stored at ultralow temperature for subsequent quantitation of transcripts levels and carotenoid accumulation.

The expression levels of *CaNAC81*, *PSY, BCH* and *CCS* genes in fruits from NTC, TRV2, and TRV2:*CaNAC81*-agroinfected plants were evaluated by quantitative PCR (qPCR), using the Elongation Factor alpha (*EF1*) and *Actin*-encoding genes as endogenous controls. Raw data were transformed using the ΔΔC_T_ method, with the expression levels in NTC fruits serving as a normalizer (Figure 6b). Significantly lower transcript accumulation of *CaNAC81* (*p* < 0.05) was detected in chili pepper fruits from plants treated with the TRV2:*CaNAC81* construct, confirming the silencing effect. Additionally, the expression of *PSY* was significantly lower (*p* < 0.01) in TRV2:*CaNAC81* fruits compared to NTC fruits, suggesting an effect on *PSY* expression as a consequence of the reduced *CaNAC81* transcript levels. Regarding the expression of *BCH* and *CCS,* analysis of variance (ANOVA) revealed no statistically significant differences in expression values among the different treatments, indicating that a decrease in *CaNAC81* transcription levels did not significantly affect the expression of *BCH* and *CCS*.

Furthermore, the carotenoid composition in fruits was quantified by High-Performance Liquid Chromatography (HPLC). Carotenoids were extracted independently from three fruits (biological replicates) from NTC, TRV2, and TRV2:*CaNAC81*-agroinfiltrated plants, and the levels of phytoene, lycopene, lutein, β-carotene, zeaxanthin, capsanthin, and capsorubin were quantified.

Representative chromatograms illustrating the separation of carotenoids by HPLC are presented in Supplementary Figure S3. In all analyzed samples, peaks with absorption spectra corresponding to lutein and lycopene were not identified at the retention times observed in the standard curves, suggesting the absence of these pigments in the samples. Figure 6c displays the accumulation profiles of pigments in the pericarp of analyzed chili pepper fruits. NTC and TRV2-infected plants exhibited similar accumulation profiles, with higher amounts of capsanthin and β-carotene, indicating that the agroinfiltration process did not affect the concentration of these compounds. In fact, fruit samples from plants agroinfiltrated with TRV2 showed significantly higher levels of β-carotene (*p* < 0.05). Interestingly, the concentrations of capsanthin and zeaxanthin were significantly lower (*p* < 0.01) in TRV2:*CaNAC81* fruits. Regarding phytoene accumulation, the corresponding absorbance peak was not detected in the chromatogram of one biological replicate from TRV2:*CaNAC81*; nevertheless, no statistically significant difference was observed compared to the other treatments. Finally, capsorubin was detected in only one biological replicate of fruits from plants treated with the TRV2:*CaNAC81* construct, indicating very low or no accumulation of this xanthophyl in the other two replicates.

Collectively, these results indicate that CaNAC81 functions as a transcriptional regulator of carotenoid biosynthesis, probably interacting with the *PSY* gene promoter. Yeast One-Hybrid (Y1H) system experiments are currently underway to investigate whether CaNAC81 interacts with *cis*-acting elements within the *PSY* gene promoter.

## 3. Discussion

The non-climacteric chili pepper (*C. annuum*) is significant horticultural crop known for accumulating a distinct array of carotenoids, particularly capsanthin, in its ripened fruits [8]. Beyond their biological roles in plants, carotenoids are important nutraceutical compounds with notable antioxidant, anti-inflammatory, antibacterial, antiviral, and anticancer properties [41]; however, our understanding of how the expression of genes encoding carotenogenic enzymes is regulated in chili pepper fruits remains limited.

### 3.1. Selection and Characterization of CaNAC81 TF Gene

As previously mentioned, our prior analyses, including co-expression studies and gene function network construction, guided our selection of *CaNAC81* as a compelling candidate for functional analysis to assess its potential role in regulating the expression of genes encoding carotenogenic enzymes. Furthermore, the evaluation of differential expression patterns across 38 genes related to the biosynthesis of carotenoids in chili pepper fruits revealed a positive correlation between the expression pattern of the *NAC* TF gene *Capana09g000936* (identified as *CaNAC81* in this study) and those of *CaPSY3*, *CaBCH*, and *CaCCS* genes [35]. This observation further supports our hypothesis that this TF acts as a regulator of the carotenoid biosynthesis pathway in chili pepper. Nevertheless, the identified orthologous of *CaNAC81* in *Solanum lycopersicum*, *SlNAC90*, which encodes an NAC TF known as Jasmonic Acid 2 (JA2) and its homolog JA2-like (JA2L), has been implicated in the regulation of stomatal movement (closure and reopening) triggered by pathogens [42].

The transcript levels of *CaNAC81* were also quantified using qPCR (Figure 2b), confirming an increase in its expression at 50 DAA, consistent with the previously observed normalized expression patterns from RNA-Seq data. Even though the expression pattern at 60 DAA of RNA-Seq and qPCR did not match exactly, it is important to mention that qPCR data represent a “trend” expressed as fold change, while RNA-Seq analyses proceed from millions of reads and are more reliable. Nevertheless, levels of accumulation of transcripts at 60 DAA were significantly higher than those in flower (0), and in fruits at 10, 20, 30, and 40 DAA. Additionally, given the association of the *CaNAC81* ortholog in tomato with stomatal movement, we quantified its expression levels in leaf, stem, and root tissues of chili pepper plants. Our results revealed a slight increase in the transcript accumulation in these tissues (Figure 2b); however, these changes were not statistically significant compared to the expression levels recorded in flowers or fruits from 10 to 40 DAA. Thus, the highest levels of *CaNAC81* expression were specifically found only in fruits of the chili pepper at 50–60 DAA. Additionally, anatomic and micromorphological studies conducted in the epidermis of *C. annuum* fruits have revealed the absence of stomata on the pericarp, they were only located at the fruit pedicle, suggesting that CaNAC81 TF might play divergent roles in climacteric and non-climacteric fruits [43].

According to a genome-wide analysis of the *NAC* TF gene family in chili pepper, *CaNAC81* is located on chromosome 9 and belongs to group I, subgroup *AtNAC3*, phylogenetically clustering with *CaNAC72*, *ANAC055* and *ANAC072*. Gene prediction analysis of *CaNAC81* confirmed the presence of two introns and a deduced amino acid sequence of 350 residues, as previously reported in [36]. Furthermore, the presence of seven motifs within the NAC domain of *CaNAC81* was consistent with earlier findings, where subgroups A and C exhibited two consensus motifs each [36].

Regarding the CaNAC81 protein structure, previous crystallographic studies of the three-dimensional conformation of NAC domains from *Arabidopsis* ANAC TF revealed that they lack the canonical helix–turn–helix motif typical of TFs. Instead, the NAC domain structure consists of a twisted β-sheet surrounded by a few helical elements [44]. Nevertheless, our prediction of the secondary and 3D structure of CaNAC81 showed a distinct conformational pattern in the NAC domain (one α-helix and six β-sheets) and a predominantly coiled structure in the TAR domain (Figure 4c,d). Although the NAC domain is generally considered a relatively conserved region, some variation between NAC subgroups, such as the presence of additional motifs or changes in subdomain structure, is expected due to differences in amino acid sequence, as indicated by a genome-wide analysis [36]. Consequently, some structural variations are also anticipated. While the in silico-generated 3D structure provides an approximate model of the CaNAC81 structure and acknowledging that the quality estimators did not reach the target scores for considering it an excellent predictor of the three-dimensional structure, this homology model, based on a reliable template, allows the visualization of the spatial conformation of this TF. It should also be considered that quality estimators like GMQE, QMEAN, and Ramachandran plots assess the complete model; therefore, we could expect higher quality results if only the NAC domain, being the DNA-binding domain with a more conserved structure across species [34], were selected for modeling.

Conversely, the analysis of the *CaNAC81* promoter revealed putative binding sequences for only four transcriptional regulatory elements: W-box, ABRE, LTR and GARE (Figure 4e), out of the all elements previously reported in [36]. This observation is consistent with the documented significant variation in the localization of transcription factor binding within the promoter of genes encoding NAC TFs.

MSA and phylogenetic analyses (Supplementary Figure S2) revealed that CaNAC81 is not an ortholog of previously reported NAC TFs as participants in the regulation of carotenoid biosynthesis in tomato, and it is not phylogenetically close to CA12g04950 TF from chili pepper. As has been described in [36], CaNAC81 and CaNAC72 TFs were consistently clustered in the same group; they were grouped together in our phylogenetic tree but evidently corresponded to different proteins.

### 3.2. Virus-Induced Silencing Assays of CaNAC81 in Chili Pepper

The involvement of CaMYB306 [23], CaBBX20 [24], CA12g04950 [15], and recently the MADS RIPENING INHIBITOR-DIVARICARA1 module genes [45] in regulating carotenoid biosynthesis in chili pepper fruits has been established through VIGS assays. Similarly, we evaluated the role of CaNAC81 as a regulator of carotenoid biosynthesis in the present study. Color changes (yellow-orange spots) were observed in fruits from plants where CaNAC81 was silenced, in contrast to the red fruits of the NTC group (Figure 5d and Figure 6a), suggesting a regulatory function of CaNAC81 in carotenoid accumulation. Significantly lower levels of CaNAC81 gene expression were detected in the silenced fruits compared to those from the NTC plants (Figure 6b). Furthermore, the transcript accumulation levels of PSY were significantly lower in fruits from plants agroinfiltrated with TRV2:CaNAC81, indicating that the reduced expression of CaNAC81 in silenced fruits led to lower expression of PSY. In contrast, no statistically significant changes were observed in the transcript accumulation levels of BCH and CCS, suggesting that the expression of these genes was not affected by silencing CaNAC81. Even though the promoters of *BCH* and *CCS* possess putative binding sites for CaNAC81, functional redundancies with other TFs could explain this phenomenon. Additionally, the VIGS technique induces only a decrease in the expression levels of a target gene; it is not a gene knock-out assay, and the silencing effect can be variable. It is important to note that the high standard deviation (SD) values associated with the relative expression levels depicted in the graphs were likely influenced by the considerable biological variability among the replicates, resulting in varying degrees of silencing. These results align with a previous report that, using stringent parameters in co-expressions (min.fdr = 0.1, min.r2 = 0.6, and n.min.acc = 12), suggested CaNAC81 (reported as NAC72) as a potential regulator of PSY [21]. Moreover, eight putative union sites for CaNAC81 were identified in the PSY promoter (Figure 3).

Phytoene synthase (PSY) has been described as the key regulatory enzyme in the carotenoid biosynthesis pathway [16]. Furthermore, in chili pepper fruits, silencing the *CaPSY1* gene resulted in pericarp color modifications due to decreased zeaxanthin content [46]. Consistent with this, we quantified lower concentrations of zeaxanthin and capsanthin in fruits from plants agroinfiltrated withTRV2:*CaNAC81*. Additionally, capsorubin was detected in only one of the three phenotypically altered fruits evaluated, suggesting that the reduction in *CaNAC81* expression levels affected the accumulation of xanthophylls in chili pepper fruits, which aligns with the observed color changes. Indeed, it has been proposed that the color variation in *Capsicum* fruits is associated with differential expression profiles of *PSY*, *LCYB*, *BCH*, and *CCS* genes [47]. Regarding phytoene, the direct metabolic product of PSY (Figure 1), no significant changes were observed in its concentration compared to NTC fruits, despite the high standard deviation. This was likely because phytoene was quantified in only two biological replicates, with only traces (not quantifiable) detected in the third fruit. Therefore, due to the high biological variability, we cannot definitively conclude whether there was a decrease in the phytoene content caused by the partial silencing of *CaNAC81*. On the other hand, while the concentration of β-carotene was significantly lower (*p* < 0.05) in silencing fruits compared to fruits from plants infiltrated with the empty TRV2 vector, no significant changes were recorded compared to NTC fruits, making the effect of *CaNAC81* silencing on the accumulation of this pigment unclear (Figure 6c). Nevertheless, the collective in silico and experimental evidence presented in this study allow us to propose that the *CaNAC81* TF plays a role in regulating carotenoid accumulation in chili pepper fruits. This paper represents the second transcription factor belonging to the NAC family reported as being involved in the accumulation of carotenoids in chili pepper fruits, and validates the selection of TF candidates performed through coexpression analyses and functional networks, which suggested that CaNAC81 constituted a strong candidate as a regulator of the carotenoid biosynthetic pathway, simplifying the election of TFs for functional assays. These results make it possible to develop experimental strategies such as CRISPR/Cas9, Yeast one-hybrid, Yeast two-hybrid, and overexpression, among other techniques that allow not only to establish the carotenoid biosynthetic genes that could be regulated by CaNAC81 but also whether this TF participates in other functions such as biotic or abiotic stress in chili pepper plants.

## 4. Materials and Methods

### 4.1. Selection and Characterization of the CaNAC81 TF Gene

The encoding and protein sequences of the CaNAC81 TF previously identified as NAC 72 (NCBI identifier XP_016569664.2) were obtained from transcriptome sequencing performed on 12 cultivars of *C. annuum*, as previously described in [21,22]. The sequence was analyzed using the Pepper Genome Platform (PGP) http://peppergenome.snu.ac.kr/blast.php (accessed on 7 November 2024) and compared with the chili pepper NAC transcription factor family, as previously described [36]. The expression profile of *CaNAC81* and target genes was visualized using the R package *Salsa* and presented as a standardized expression profile, according to [38]. The analysis of the target gene (TG) promoter regions was conducted as reported in [21], using PlantPAN 4.0 software https://plantpan.itps.ncku.edu.tw/plantpan4/index.html (accessed on 23 January 2025) [48].

The genomic sequence of *CaNAC81* was obtained from the National Center for Biotechnology Information (NCBI), and the open reading frame (ORF) sequence was corroborated using the AUGUSTUS server (Version3.3.3) https://bioinf.uni-greifswald.de/augustus/submission.php (accessed on 17 October 2024) [49]. Prediction of protein domains and identification of important protein sites were performed using the InterPro tool from EMBL’s European Bioinformatics Institute (EMBL-EBI) database https://www.ebi.ac.uk/interpro/ (accessed on 17 October 2024) [50]. The presence of conserved motifs characteristic of the NAC domain was corroborated using the Motif Alignment and Search Tool (MAST) version 5.5.7 of The MEME Suite https://meme-suite.org/meme/ (accessed on 25 October 2024) [51]. The organization of motifs into five characteristic subdomains (A-E) of the NAC family was based on previously published alignments [25,36]. The search for *CaNAC81* orthologs in *Solanum lycopersicum* and *A. thaliana* was conducted using the Plant Transcription Factors database (PlantTFDB) v5.0 https://planttfdb.gao-lab.org/index.php (accessed on 29 October 2024) [52].

The secondary structure prediction of the CaNAC81 TF was performed using the Jpred 4 Server https://www.compbio.dundee.ac.uk/jpred4/index_up.html (accessed on 10 February 2025) [53], and the three-dimensional model was predicted using the SWISS-MODEL automated protein structure homology-modeling server https://swissmodel.expasy.org/ (accessed on 10 January 2025) [54]. Template searches were conducted using the Basic Local Alignment Search Tool (Blast+) [55] and HHblits [56] against the SWISS-MODEL template library. The CaNAC81 3D model was built based on the target-template alignment using ProMod3, and model quality estimation (global and per-residue) was evaluated using the QMEAN scoring function [57]. Energy minimization of the model was performed using Anolea and GROMOS96, as implemented in the Swiss PDB Viewer (SPDBV 4.01) [58].

The promoter region of *CaNAC81* was analyzed by selecting 2500 pb upstream of the ATG start codon and located on the *VYZY01017293* locus of *C. annuum* cultivar ECW scaffold192042. The localization of the TATA box, Initiator element (INR), and Transcription Start Site (TSS) was predicted using the YAPP Eukariotic Core Promoter Predictor https://www.bioinformatics.org/yapp/cgi-bin/yapp.cgi (accessed on 17 October 2024). The search for putative binding sites of the 11 regulatory elements previously reported in [36] was conducted using the New PLACE database https://www.dna.affrc.go.jp/PLACE/?action=newplace (accessed on 23 January 2025) [59].

Finally, phylogenetic analyses of CaNAC81 and other NAC TFs from tomato and chili pepper previously reported as regulators of carotenoid accumulation have been performed. Protein sequences were aligned using the Clustal Omega tool from EMBL-European Bioinformatics Institute https://www.ebi.ac.uk/jdispatcher/msa/clustalo (accessed on 22 January 2025) [60]. Construction of phylogenetic trees was conducted by Neighbor-Joining and Maximum Likelihood tests using Mega X package [61] using *p-distance* and WAG substitution, respectively. Branch support values were estimated via bootstrap analysis of 500 replicates.

### 4.2. Plant Growth Conditions

To obtain flowers and fruits at different growth and ripening stages, as well as leaf, stem and root tissues of chili pepper, seeds of *C. annuum* cv. Tampiqueño 74 were germinated and maintained under greenhouse conditions until they reached the adult stage. Plants were supplemented every two weeks with a solution of N:P:K 30:20:10 (FerviaFol of Agroquímicos Rivas). Fruits at 10, 20, 30, 40, 50, and 60 DAA were collected and dissected. The pericarp was separated from the placenta and seeds, then immediately frozen and preserved at ultralow temperature for total RNA purification. Flowers, leaves, stems and roots were collected, washed with sterile water, and then frozen and preserved under the same conditions as the fruits.

For the silencing assays, seeds of *C. annuum* ‘Tampiqueño 74’ were germinated and cultivated in a growth chamber at 28 °C, with a relative humidity of 66%, and a 16-h light photoperiod at a photon flux density of 70 μmol/m^2^/s^2^ using T8W/Starcoat GE fluorescent lamps. Chili pepper plants were fertilized as described previously. Germination and growth conditions were performed according to [62].

### 4.3. Virus-Induced Silencing of the CaNAC81 Gene

Previous to primer design for VIGS vector constructions, no off-target region corresponding to nucleotide 591 to 960 was identified in the *CaNAC81* sequence. This analysis was performed using the *pssRNAit* web server https://www.zhaolab.org/pssRNAit/ (accessed on 23 January 2022) [63]. Then, a 410 pb fragment of *CaNAC81* (nucleotide 505 to 914) was amplified using the primer pair detailed in Supplementary Table S3. Restriction sites for *XbaI* and *SacI* were incorporated into the forward and reverse primers, respectively, for the construction of the viral silencing vectors. Amplification reactions were performed using the *Taq* DNA Polymerase Recombinant kit (Invitrogen, Carlsband, CA, USA) following the manufacturer’s instructions. The resulting amplification products were ligated into the pCR 4-TOPO Vector (Invitrogen, Carlsband, CA, USA) and cloned into the One Shot TOP 10 chemically competent *Echerichia coli* cells.

The pTRV1 and pTRV2 vectors were employed for VIGS silencing assays. The *CaNAC81* gene fragment was obtained by enzymatic digestion of plasmids purified from *E. coli* and ligated into the pTRV2 vector using T4 DNA ligase, following the manufacturer’s instructions. *E. coli* DH10B competent cells were transformed with the recombinant plasmids. Positive clones were selected and confirmed by purifying the recombinant plasmids and performing restriction digestions. Electrocompetent cells of *Agrobacterium tumefaciens* GV2260 were transformed with pTRV2:*CaNAC81*. Agroinfiltration of chili pepper seedlings was performed according to [62]. The pTRV2:*PDS* construct served as a positive control of agroinfiltration and gene silencing, while plants agroinfiltrated with pTRV2 (empty vector) or infiltrated with sterile distilled water were used as negative controls (NTC).

Fruits exhibiting phenotypic variations were collected at 50–60 DAA. Pericarp tissue was harvested, immediately frozen in liquid nitrogen, stored at −80 °C and used for quantitative PCR (q-PCR) and HPLC analyses.

### 4.4. CaNAC81 Expression Analysis by q-PCR

Total RNA was isolated from 100 mg of frozen pericarp or chili pepper plant tissues using TRIzol Reagent (Invitrogen) and the PureLink RNA MiniKit (Invitrogen, Carlsband, CA, USA). RNA samples were treated with DNase I according to the manufacturer’s instructions (Invitrogen). The quality and concentration of total RNA preparations were assessed using a NanoDrop 2000c Spectrophotometer (Thermo Scientific, Asheville, NC, USA). One microgram of total RNA was used for cDNA synthesis with the SuperScript III First-Strand Synthesis System kit (Invitrogen).

A 182 pb fragment of cDNA) was amplified by qPCR with specific primers designed for the *CaNAC81* gene (nucleotides 40-63 and 198-221, respectively), as detailed in Supplementary Table S3. Fragments of genes encoding Elongation Factor alpha (*EF1*) and Actin, as reported in [64], were used as internal control genes. Additionally, the expression of *PSY*, *BCH* and *CCS* genes was evaluated in fruits exhibiting carotenoid-related phenotypic changes using primer pairs previously reported in [65] (Supplementary Table S3). The specificity of the *CaNAC81* forward and reverse primers designed for qPCR assays was verified by melting curve analysis, and primer efficiency was verified using a standard curve. The primers showed a slope of −3.43 and R^2^ of 0.99, with an efficiency of 95.36%. Reactions were performed in a total volume of 10 μL containing 200 ng of cDNA, 2 pM of forward and reverse primers, and 5 μL of Fast SYBR Green Master Mix (Applied Biosystems, Asheville, NC, USA). No-template controls were included in all plates. The qPCR assays were performed in MicroAmp Fast Optical 48-Well Reaction Plates (Applied Biosystems) sealed with MicroAmp Optical Adhesive Film (Applied Biosystems) on a StepOne Real-Time PCR System (Applied Biosystems) under the following conditions: 95 °C for 10 min, followed by 40 cycles at 95 °C for 40 s, the specific annealing temperature for each primer pairs (indicated in Supplementary Table S3, and 72 °C for 40 s, with a melting curve at 95 °C for 15 s, 60 °C for 1 min, and 95 °C for 15 s.

Relative quantification of gene expression was evaluated using the comparative 2^−ΔΔC^_T_ method according to [66]. Data were obtained from three biological replicates, each run-in triplicate, and analyzed using Excel. Analysis of variance (ANOVA) and a post hoc Tukey test were performed using R version 4.0.0. The results are reported as relative expression levels ± standard deviation (SD).

### 4.5. Separation and Quantification of Carotenoids by HPLC

The carotenoid content in fruits of 50–60 DAA exhibiting phenotypic changes was evaluated using High-Performance Liquid Chromatography (HPLC). The pericarp of three biological replicates was independently frozen and lyophilized for 24 h. Carotenoid extraction and saponification from 250 mg of pericarp tissue were performed according to [67]. Carotenoid separation was carried out using a YMC carotenoid column (4.6 × 250 mm, 5 μm particle size) coupled to an HPLC (series 1200, Agilent Technologies). Twenty microliters (20 μL) of each sample were injected and separated following the manufacturer’s recommended method, using methanol/methyl *tert*-butyl ether (MTBE)/water (81:15:4) as mobile phase A and methanol/MTBE/water (7:90:3) as mobile phase B, with a run time of 55 min, a flow rate of 1 mL/min, and a column temperature of 25 °C. A standard curve with six concentration points was prepared using capsorubin (CaroteNature, Ostermundigen, Switzerland). Capsanthin, lutein, zeaxanthin, β-carotene, and phytoene were acquired from Sigma-Aldrich, Merck, St. Louis, MO, USA, EE. UU. Carotenoids were detected at 450 nm, and phytoene was detected at 285 nm.

Data were obtained from three biological replicates, and the concentration of each carotenoid was estimated using the standard curve and the formulas reported in [67]. Carotenoid concentrations are reported as milligrams per gram dry weight (mg/g DW) ± SD. Statistical significance was evaluated using ANOVA and a post hoc Tukey test in the R version 4.0.0 statistical software package.

## 5. Conclusions

In this article, we have provided a detailed explanation of why *CaNAC81* TF represents a strong candidate to regulate the carotenogenic pathway in chili pepper fruits. Furthermore, the characterization of the *CaNAC81* gene, its expression pattern corroborated by RNA-Seq and qPCR methods, and the analysis of the protein structure and promoter region further support and expand the available information on this TF. Additionally, the function analysis of *CaNAC81*conducted in chili pepper plants, along with the evaluation of gene expression levels and carotenoid accumulation in fruits from silenced plants, provides a solid foundation for suggesting that the *CaNAC81* gene is a transcriptional regulator of the carotenoid biosynthetic pathway in chili pepper fruits, probably through interaction with the *PSY* gene promoter.

## Data Availability

The original contributions presented in this study are included in the article/Supplementary Material. Further inquiries can be directed to the corresponding author.

## Supplementary Materials

The following supporting information can be downloaded at https://www.mdpi.com/article/10.3390/plants14142099/s1: Figure S1: Sequence of *CaNAC81*; Figure S2: Multiple sequence alignment and phylogenetic tree of NAC transcription factors. Figure S3: Representative chromatograms of HPLC chili pepper fruit carotenoid separation; Table S1: Localization of putative binding sites of CaNAC81 TF on the promoter region of carotenoid-biosynthesis-related genes; Table S2: Localization of putative binding sites to regulatory elements on the promoter region of *CaNAC81* gene; Table S3: Primer pairs used in this study.
