# Supplementary material for: The Transcription Factor CaNAC81 Is Involved in the Carotenoid Accumulation in Chili Pepper Fruits
Source: Plants (Basel). 2025 Jul 8;14(14):2099. doi: 10.3390/plants14142099 (PMC12298505; doi:10.3390/plants14142099)
Supplement: Supplementary file 1 [file plants-14-02099-s001.zip › plants-3683601-supplementary-re1/FigS1.pdf]

Sequence of *CaNAC81*

ATG GGT GTT CAA GAA AAA GAT CCA CTT TTG CAA TTA AGT TTG CCA CCA GGG TTT AGA TTT
M G V Q E K D P L L Q L S L P P G F R F
TAT CCG ACT GAT GAA GAG CTT TTA GTT CAA TAT TTG TGT AAG AAA GTT GCT GGC CAT AAT
Y P T D E E L L V Q Y L C K K V A G H N
TTT CCT CTA CAA ATT ATT GGA GAA ATT GAT TTG TAC AAA TTT GAT CCT TGG GTT CTA CCT
F P L Q I I G E I D L Y K F D P W V L P
agt aagtttctagttcaatcttcttttggaatttttgctcagtatctgatattgaatcgaggcttgtttaattcaaatt
S
tgcgtttagcttatcgaaattgagacctctagttaataatgaacgaattctatctgtcacaccgtaatccttgttggtatt
actttgtgatattggaccgatcaaaaggaagagagtttttttatagtcttaaatgtatcattatatttttctagctttaa
aaatatgtcattaagggtaataaaaaatctaaagtcaaattatttttagagtgtagcgattgagatgcttttaatgaacta
ataagaaaatagtgttatataaaatgaaacactaatttaggtattcgaatttgaatgaggttggaatttatggggaaaaaa
gggcttttgaaacttgtaaattacgcatacaatgagatatttgtagctttggaagtttgccactacgactaacaggaaatg
ttaaattaattttgactcaattcttcaccaattatttttaaattatatttttgtattattaattatttgaatttgttaatt
gatgatttttttcgtgtttgatttgttataggt AAG GCA ATG TTT GGA GAA AAA GAA TGG TAC TTT TTT
K A M F G E K E W Y F F
AGT CCA AGA GAT AGG AAG TAT CCA AAT GGA TCT CGA CCC AAT AGA GTT GCC GGG TCG GGT
S P R D R K Y P N G S R P N R V A G S G
TAT TGG AAA GCA ACG GGT ACT GAT AAG GTT ATA ACA TCA CAA GGG AGG AAA GTT GGA ATT
Y W K A T G T D K V I T S Q G R K V G I
AAG AAA GCG CTT GTG TTT TAT GTT GGT AAA GCA CCA AAA GGA TCC AAG ACT AAT TGG ATC
K K A L V F Y V G K A P K G S K T N W I
ATG CAT GAA TAT AGA CTT TTT GAA ACT TCA AGG AAA AAT GGA AGT TCA AAG gtaatttcacatt
M H E Y R L F E T S R K N G S S K
actcctttcttttttttttttcacgaaaatttgcacttttaattcctcaattattcataaattttagtacttatgatctt
tttaagtacatttaaccttcaatcaactaaaatgtgcactttcgactcactttcttgtgaatctttgcaaattaagtgaat
tattaaaaataagccataaaaatttatgtggttattgaacatttcattaggggtaaaatgagtagtttgaagttaaattgt
cattgaatgtgaaaaaatgtcccttatttttttatggaataaaacgaaaatagtcgcataaagtcagtgcaagaaagggtg
ttacataaatcgggccgggaggggaggggtgggtggttctatacgatgaatttgtacctttgcacctggtatgcttctata
tgttaaaagtaatttggttaatctggaaaaaagtgtaaatagaacatgttttgtataataaagggaccaggaatgtacatt
ttaattgattggaggttaacatgactttggctcaacggccaaagtcaaaattcacaataactggaagaccaaatgtgcaat
tctacctcattttcaattgtctggttgcaattcttaatgtgtatatggtgtttgtatatttcag CTA GAT GAA TGG
L D E W
GTG TTA TGT CGA ATT TAT AAG AAG AAT TCA AGT GGA CCA AAA CCT CTT ATG CCT GGT TTA
V L C R I Y K K N S S G P K P L M P G L
CGC AGC AAT GAA TAC AGC CAT GCT GCT TCT TCG ACG ACT TCT TCT TCA TCC CAA TTC GAT
R S N E Y S H A A S S T T S S S S Q F D
GAT ATG CTC GAA TCA TTA CCA GAA ATG GAT GAT CGG TTT TCT AAC TTG CCA AGA TTG AGC
D M L E S L P E M D D R F S N L P R L S
TAT CTT AAA ACC GAA AAA CTG AAC CTC GAA CGC CTG GAT TCA GCC AAT TTT GAT TGG GCG
Y L K T E K L N L E R L D S A N F D W A
ATA CTC GCA GGG CTC AAA CCA ATG CCG GAA TTG GGC CCT TCA AAT CAA GCT CCA GGC GTT
I L A G L K P M P E L G P S N Q A P G V
CAC GCC CAG GCT CAG GCG CAG GTC AAC AAC CTC ATC CAG AAC AAC AAT AAT ATG AAT TTT
H A Q A Q A Q V N N L I Q N N N N M N F
CTG AAT GAT GTT AAT GCT CCT CCT ACG AAT ATC AGA AGC AAC ACC AAG GTT GAA AGT ATT
L N D V N A P P T N I R S N T K V E S I
AAT CTG GAC GAA GAA GTT GAA AGC GGA ATC AGG AAT CAA CGG TTT GAT AAA TCA GGT TAC
N L D E E V E S G I R N Q R F D K S G Y
TTC CAA CAG AAT ATG AAT GGA CTT TCT CAA TTG TAC ACG AAC AAT GTC AAT GTC GGC CAA
F Q Q N M N G L S Q L Y T N N V N V G Q
TTC GGA ATC CAG TGT CCG AAC CAG ACA TTG AAT CTG GGA CTC AGG CAG TAG
F G I Q C P N Q T L N L G L R Q *

Exon1: 1-183
Exon 2: 779-1045
Exon 3: 1690-2292
Subdomain A
Subdomain B

Subdomain C
Subdomain D
Subdomain A
Transcriptional Activation Region (TAR)
