# Supplementary material for: The Transcription Factor CaNAC81 Is Involved in the Carotenoid Accumulation in Chili Pepper Fruits
Source: Plants (Basel). 2025 Jul 8;14(14):2099. doi: 10.3390/plants14142099 (PMC12298505; doi:10.3390/plants14142099)
Supplement: Supplementary file 1 [file plants-14-02099-s001.zip › plants-3683601-supplementary-re1/FigS2.pdf]

(a)

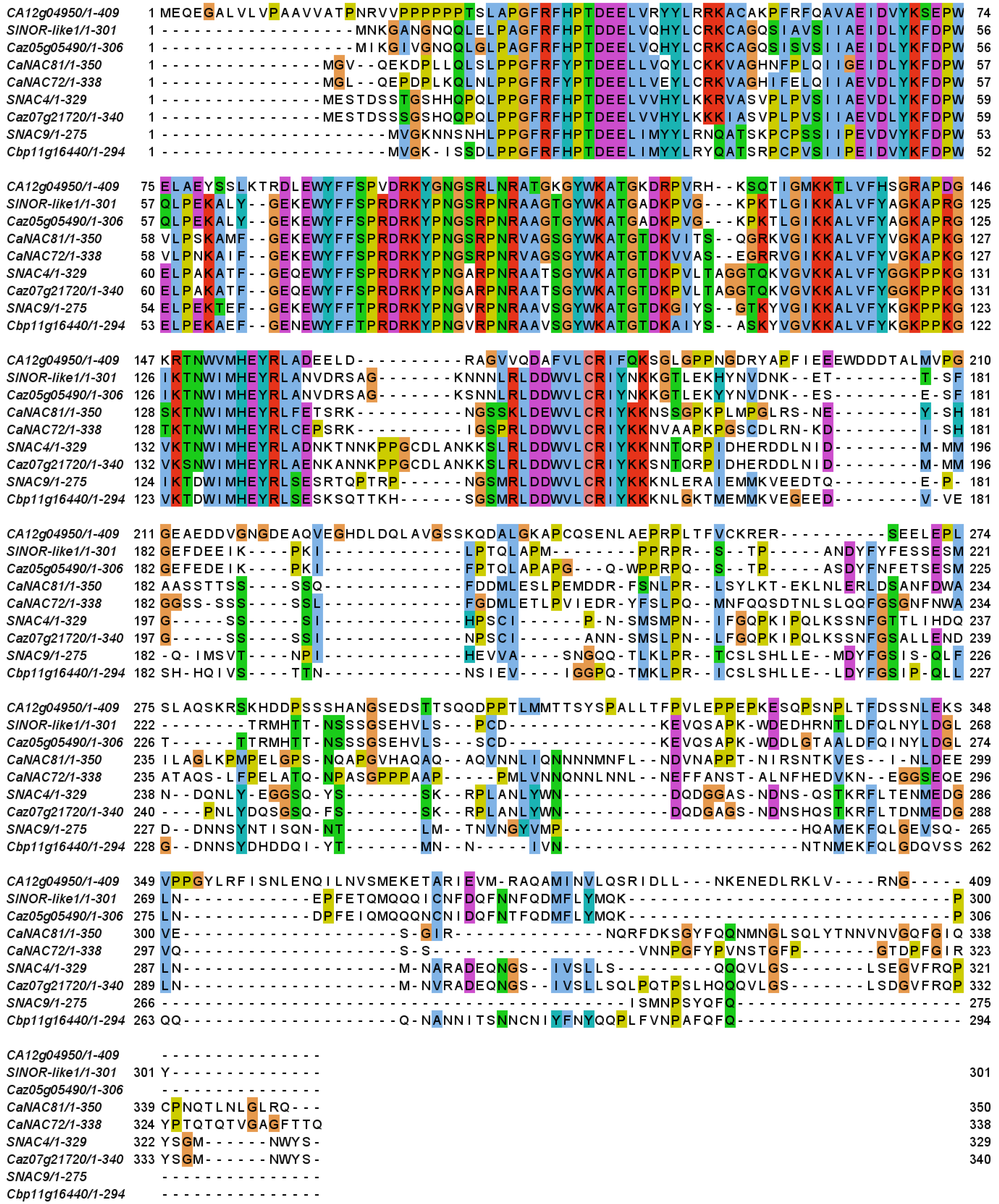

(b)

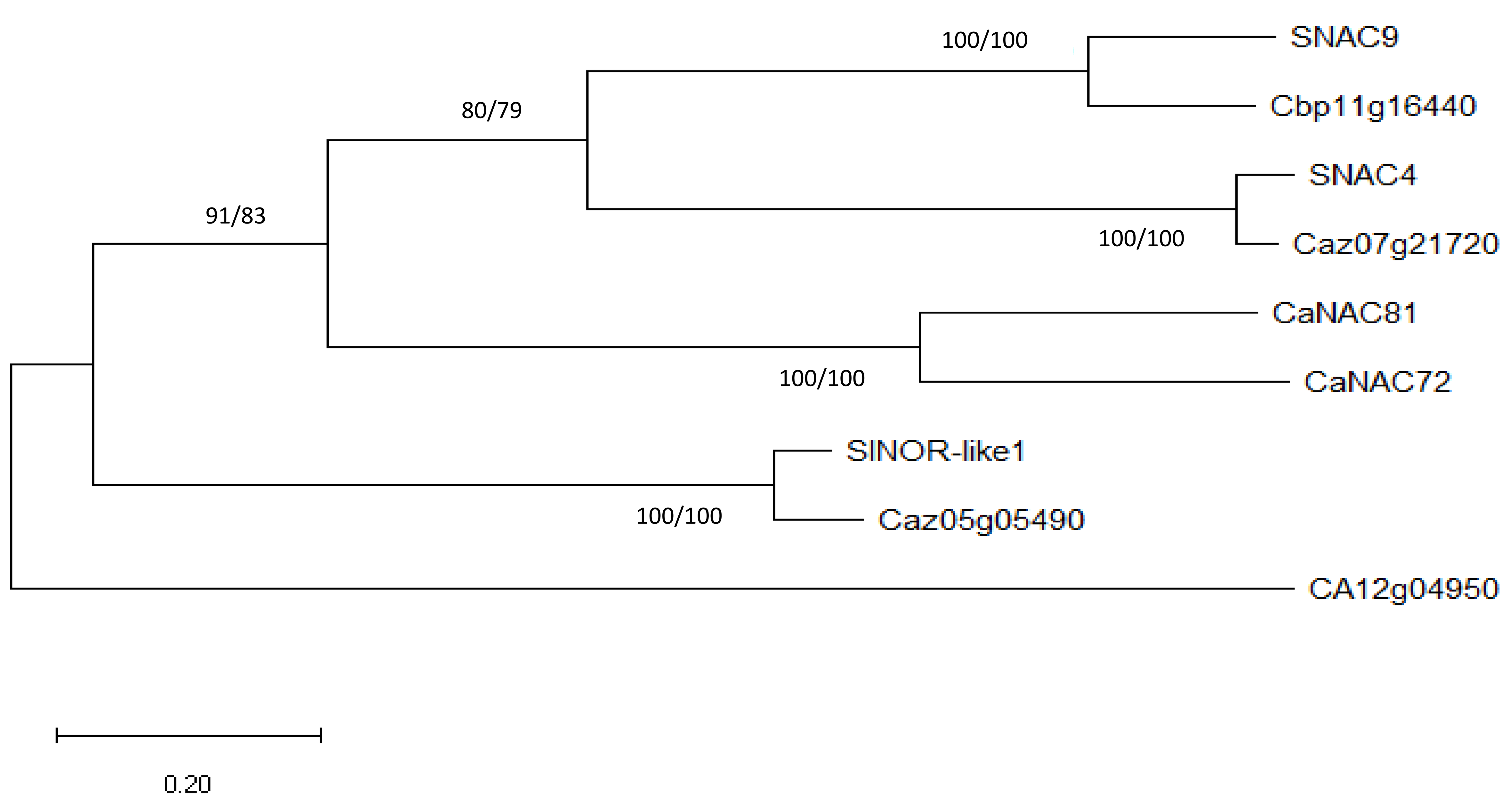

NAC TF	Fruit	Reference
SNAC4	*Solanum lycopersicum*	[39]
SNAC9	*Solanum lycopersicum*	[39]
SlNOR-like 1	*Solanum lycopersicum*	[40]
Cbp11g16440	*C. baccatum var. pendulumC. pubescens*	[37]
Caz07g21720	*Capsicum annuum var. annuum*	[37]
Caz05g05490	*Capsicum annuum var. annuum*	[37]
CA12g04950	Pepper 'Honglong 23'	[15]
CaNAC81	*Capsicum annuum*	This study
CaNAC72	*Capsicum annuum*	[36]
