# Supplementary material for: The Transcription Factor CaNAC81 Is Involved in the Carotenoid Accumulation in Chili Pepper Fruits
Source: Plants (Basel). 2025 Jul 8;14(14):2099. doi: 10.3390/plants14142099 (PMC12298505; doi:10.3390/plants14142099)
Supplement: Supplementary file 1 [file plants-14-02099-s001.zip › plants-3683601-supplementary-re1/FigS3.pdf]

Negative control. Sample B62

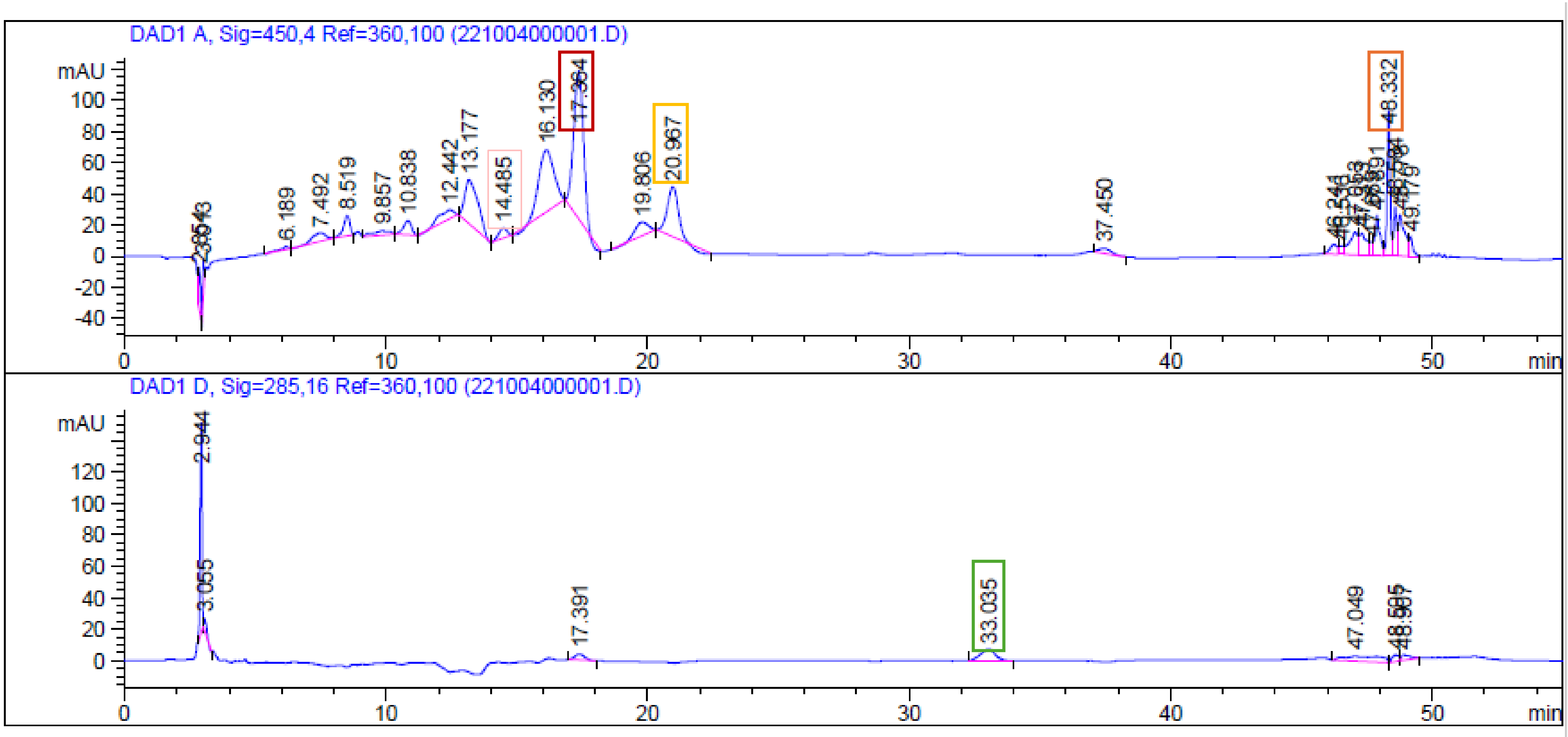

Capsorubin

Capsanthin

Zeaxanthin

β-carotene

Phytoene

TRV2. Sample B89

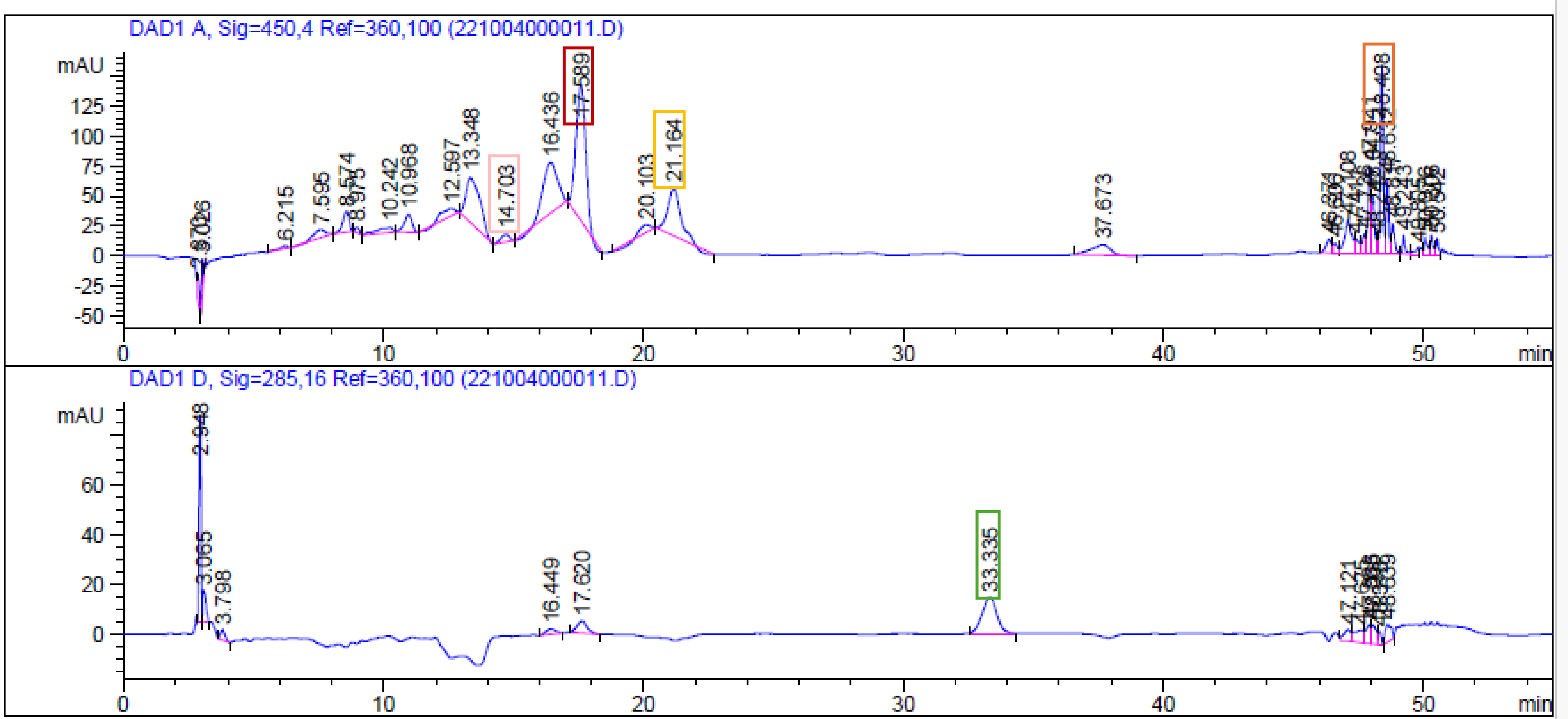

Capsorubin | Capsanthin | Zeaxanthin | β-carotene | Phytoene

TRV2:CaNAC81. Sample B32

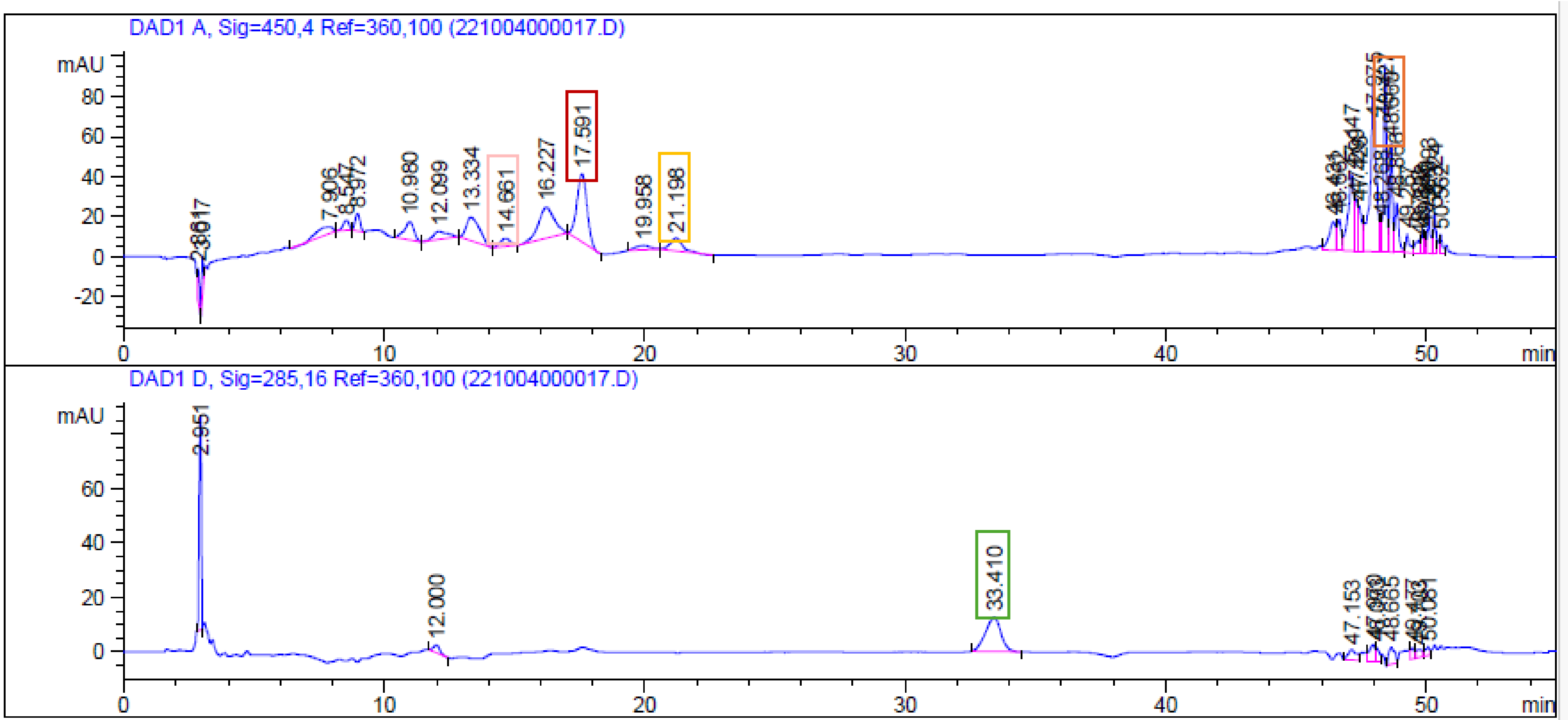

Capsorubin | Capsanthin | Zeaxanthin | β-carotene | Phytoene
